# Development of a Novel Multi-Disciplinary Specialized Care Service for Children and Adolescents with Autism Spectrum Disorder and/or Intellectual/Developmental Disability in a Tertiary Children’s Hospital Setting

**DOI:** 10.3390/children10010057

**Published:** 2022-12-27

**Authors:** Joelene F. Huber, Alvin Loh, Suneeta Monga, Jessica Esufali, Michelle Shouldice

**Affiliations:** 1 Division of Paediatric Medicine, Department of Paediatrics, The Hospital for Sick Children, Toronto, ON M5G 1X8, Canada; 2 Surrey Place, Toronto, ON M5C 2C2, Canada; 3 Division of Developmental Paediatrics, Department of Paediatrics, Temerty Faculty of Medicine, University of Toronto, Toronto, ON M5S 1A8, Canada; 4 Department of Psychiatry, The Hospital for Sick Children, Toronto, ON M5G 1X8, Canada; 5 Department of Psychiatry, Temerty Faculty of Medicine, University of Toronto, Toronto, ON M5S 1A8, Canada; 6 Division of Paediatric Medicine, Department of Paediatrics, Temerty Faculty of Medicine, University of Toronto, Toronto, ON M5S 1A8, Canada

**Keywords:** autism spectrum disorder, Intellectual/Developmental Disability, mental health, developmental pediatrics, behavior-medicine, tertiary care

## Abstract

Children and adolescents with autism spectrum disorder (ASD) and/or Intellectual/Developmental Disability (IDD) are at greater risk of developing comorbid medical conditions, mental health diagnoses, behavioral challenges, and having overall poorer physical and mental health outcomes. Hospital environments present unique stressors and challenges for children and adolescents with ASD/IDD including a change in familiar environment, unpredictable routines, and exposure to sensory stimuli that may be overwhelming. While many school boards have specialized multi-disciplinary special needs support teams and services made up of professionals with expertise in supporting students with ASD/IDD, most hospitals do not have a formal multi-disciplinary ASD/IDD support team in place to support patients, families, and health care staff across the hospital. There is an emerging recognition of the need for specialized multi-disciplinary developmental-behavioral and mental health expertise in hospital inpatient settings. This paper describes the framework for the development of an innovative multi-disciplinary program to better support children and adolescents with ASD/IDD within a tertiary children’s hospital setting.

## 1. Introduction

Children and adolescents with autism spectrum disorder (ASD) and/or Intellectual/Developmental Disability (IDD) are at greater risk of developing comorbid medical conditions [1], mental health diagnoses [1,2] and behavioral challenges. Approximately 30% of autistic individuals have IDD, physical and mental health diagnoses [3]. Children and adolescents with ASD/IDD frequently experience barriers in accessing health care, delays in diagnosis, medical care and treatment, more health care encounters, more hospitalizations, longer length of hospital stay, overall poorer physical and mental health outcomes, and may experience higher levels of stress and agitation and struggle to cope with health care encounters [1,4,5,6,7,8]. Autistic individuals [9] and individuals with IDD [6] are at greater risk of premature mortality than their same-age peers without ASD/IDD. Often, these health outcome inequities could be avoided with appropriate, specialized care [1].

Patients with ASD/IDD frequently present to the hospital emergency department when facing a medical, mental health, behavioral, and/or family/social crisis. Further, many children and adolescents with ASD/IDD have periodic and/or chronic health conditions requiring care in an emergency department setting or a medical admission in a hospital. In these settings, patients often require invasive medical procedures, tests, or treatments, and find themselves in a new and unfamiliar hospital environment. Overall, children and adolescents with ASD are hospitalized six times as often as their neurotypically developing peers [7].

Hospital environments present unique stressors and challenges for children and adolescents with ASD/IDD including a change in environment, unpredictable routines and exposure to sensory stimuli that may be overwhelming [8,10]. Research shows that children and adolescents with ASD admitted to an inpatient pediatric medical unit are at an increased risk of experiencing high levels of distress and exhibiting agitation, posing safety risks for themselves, caregivers, and healthcare staff [8,11]. Further, children and adolescents with ASD/IDD with aggressive and self-injurious behaviors are more likely to be admitted to hospital [12]. Health care professionals report they often feel poorly equipped to support patients with ASD/IDD due to a lack of training in this specialized area of medicine [8]. Both parents and health care professionals feel additional specialized expertise and training are needed to meet the individual needs of children and adolescents with ASD/IDD in health care environments [13,14]. While many school boards have specialized multi-disciplinary special needs support teams and services made up of professionals with expertise in supporting students with ASD/IDD [15], most hospitals do not have a formal multi-disciplinary ASD/IDD care team in place to support patients, families, and health care staff across the hospital, including inpatient and emergency department settings.

The prevalence of ASD has risen by over 270% in the past two decades, from 1 in 150 in 2000 to 1 in 40 in 2018 [16]. With this rising patient population, along with the high incidence of medical and mental health comorbidities that lead to increased hospitalizations, in addition to the known health inequities [1,4,5,6,7,17], it is now more crucial than ever that hospitals and health care professionals are equipped to meet the individual needs of children and adolescents with ASD/IDD [8].

There is an emerging recognition of the need for specialized hospital-based multi-disciplinary developmental pediatric expertise in inpatient, emergency department and ambulatory care hospital settings [8]. Further, hospital system-level changes, funding for additional resources, adaptations to hospital architecture and design to meet the individual needs of patients with ASD/IDD, and capacity building among health care professionals and learners are required.

Recognizing the physical and mental health inequities across the life span of individuals with a diagnosis of ASD/IDD, the Ministry of Health and Long-Term Care in Ontario, Canada, provided funding to a specialized developmental center “with a passionate commitment to improving the lives of people with ASD/IDD and their families” and to provide interdisciplinary clinical services to “help people with IDD and ASD to lead healthy and socially inclusive lives…” [18]. The purpose of this funding was to improve access and quality of care through developing better health and mental health system pathways for children and adolescents with ASD/IDD through collaborative partnerships between Developmental Pediatrics, Psychiatry/Mental Health, Pediatric Medicine, Family Medicine, and community partners; solve and improve issues in care delivery; build capacity among health care professionals and learners; decrease ableism in health care; provide inclusive and adaptive care in accessible/adaptive environments; improve transition to adult care; and serve as a model-of-care for other centers and hospitals to follow.

This paper describes the framework for the development of an innovative partnership between a specialized developmental center and a tertiary children’s hospital, that arose from the funding initiative, to collaboratively implement a multi-disciplinary program to better support children and adolescents with ASD/IDD, integrated within a tertiary hospital setting. The aim of this initiative and collaborative partnership was to develop a model of integrated multi-disciplinary hospital care to address health inequities, meet the individual needs of patients with ASD/IDD and families across the hospital, build capacity among health care professionals and learners, decrease stress and agitation associated with hospital health care encounters, improve hospital safety of patients, caregivers and staff, and ultimately improve physical and mental health outcomes of people with ASD/IDD.

## 2. Program Development

### 2.1. Stakeholder Engagement

Integrated, semi-structured stakeholder interviews were conducted virtually (as program development occurred during the pandemic) with a range of relevant stakeholders, including hospital leadership and multi-disciplinary care professionals across the hospital (including, but not limited to, psychiatry, pediatric medicine, neurology, emergency medicine, complex care, psychology, social work, rehabilitation services/allied health services, child life, inpatient services, outpatient ambulatory clinic services) and across the developmental sector, including developmental service agency partner(s) and regional developmental children’s treatment center(s).

The purpose of stakeholder engagement was to obtain the perspectives of stakeholders by identifying themes surrounding gaps in care for children and adolescents with ASD/IDD. Stakeholders were asked which patients/patient settings require support, how to deliver this support and how best to integrate specialized care within the current services in the hospital, while complementing existing developmental-behavioral and mental health services for children and adolescents with ASD/IDD across the regional developmental sector and health care system. Stakeholders were each asked the same questions (Table 1) that were used as a starting point for discussions to obtain their input and perspectives.

### 2.2. Tertiary Level Hospital Care Needs Themes for Caring for Children and Adolescents with ASD/IDD

The themes that arose from the stakeholder input regarding the needs for a specialized developmental-behavioral and mental health integrated tertiary level hospital care program for children and adolescents with ASD/IDD are presented in Table 2.

### 2.3. Program Structure

#### 2.3.1. Literature Review Recommendations for Providing Hospital-Based Medical Care to Children and Youth with ASD

The results and recommendations of the literature review published by Thom et al. [8] were used to support the development of this program. These included a literature search of the PubMed database for English manuscripts relating to hospital and inpatient medical care of children and youth with ASD using search terms including “autism spectrum disorder”, “inpatient”, “hospital”, “pediatric”, “medical”, “children”, and “adolescents”, see [8]. The recommendations in the results [8] were used in the development of this program, including: involving the patient and parents (i.e., Family-centered care); approaches to social-communication differences in the hospital setting; adaptive care strategies that are sensitive to and support the sensory needs of patients in the hospital setting; adaptive care approaches for restricted and repetitive patterns of behavior and/or interests; adaptive care approaches for procedures; proactive strategies to reduce agitation and provide supportive management of agitation and promote safety; systems-based and quality improvement intervention and capacity building; training and additional resources and human resources for pediatric hospital programs and staff [8].

#### 2.3.2. Inspiration from Models of Adaptive Care in Other Pediatric Hospitals

In addition to recommendations for program development based on the literature review [8], program design and service delivery models in other pediatric hospitals were used as a framework to build on within our own unique hospital setting, considering the current resources and needs, with a multi-disciplinary focus. These included the Adaptive Care Program at Children’s Hospital of Colorado (childrenscolorado.org) and the Adaptive Care Team service model at Cincinnati Children’s hospital (cincinnatichildrens.org) which were broadly used as examples of service models. Program conceptualization that ensued included the development of a unique inter-disciplinary care approach that included a multi-disciplinary team [(i.e., developmental pediatrician(s), child and adolescent psychiatrist(s), nurse(s), behavior analyst(s), developmental service worker(s), developmental service coordinator(s)] integrated within the point-of-care team [(i.e., general pediatrician/sub-specialty physician/surgeon(s), nurse practitioner(s), nurse(s), procedural technician(s)] and supported by established clinical programs and specialists, such as child life specialists and anesthesiologists.

#### 2.3.3. School Support Teams as a Model for an Inter-Disciplinary Consultative Paradigm

Schools and school boards have been leaders in providing specialized and individualized support for children and youth with ASD/IDD, along with providing team case conferences and consultation to teachers and school staff for many years. Specialists, including developmental pediatricians, child and adolescent psychiatrists, behavior analysts, developmental service coordinators, and general pediatricians frequently recommend referrals to school support team professionals and engage with school support team members in partnership to support shared students/patients/clients. The structure of this program drew from knowledge of school support team structures and specialized consultative school team models as well as from published models of interdisciplinary school teams to support students with ASD/IDD, i.e., [15], and such models of multi-disciplinary consultative support approaches were adapted to the pediatric hospital environment.

### 2.4. Clinical Care Pathways

A multi-disciplinary developmental-behavioral and mental health service, providing consultation for individualized and adaptive care in a tertiary level children’s hospital, was developed. The service is called the ABILITY program, which is a strengths-based acronym of words that capture the objectives of the program (Adaptive Behavioral Intellectual/deveLopmental disability Individualized & Integrated Tertiary care for Youth and children), to adapt the care we provide to meet the individual needs of patients and families in order to provide integrated tertiary level care.

The model of care was based on the input from integrated stakeholder interviews described in Table 2. Further, it incorporated strategies based on other collaborative models of care aimed at improving health care for children with ASD/IDD in the literature, i.e., [19] and based on a review of the hospital-based care needs of children with ASD/IDD described in the literature, i.e., [8]. The multi-disciplinary model of care was designed to provide consultative support to patients, families, and health care staff across the hospital, similar to a school-based multi-disciplinary support team model that provides specialized expertise to children and adolescents with ASD/IDD and their teachers [15]. The ABILITY team provides consultation wherever patients present or require support across the hospital, including: the emergency department; where procedures, test and treatments are being administered; across all inpatient units; and in outpatient clinics. The team responds urgently to emergency department and inpatient unit patients and offers scheduled outpatient consultation for patients referred to the program and for follow-up care.

**Figure 1:**
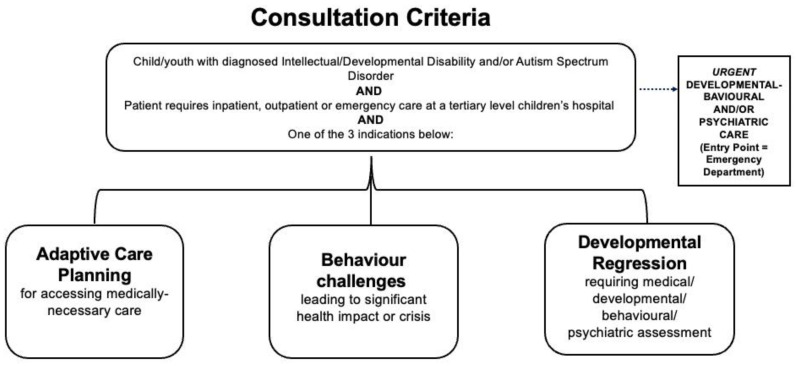
Developmental-behavioral and mental health tertiary children’s hospital service consultation criteria and clinical pathways.

The consultation criteria and clinical pathways are shown in Figure 1. Children and adolescents with a diagnosis of ASD/IDD who require tertiary level emergency department, inpatient or outpatient hospital care can be referred to the multi-disciplinary team. The service provides three main pathways of care across the hospital as shown in Figure 1.

#### 2.4.1. Adaptive Care Planning

Children with ASD/IDD often experience barriers in accessing medically necessary care, higher levels of stress and agitation in hospital settings and struggle to cope with health care encounters. As a result, they may experience delays in diagnosis, medical care and treatment, and overall poorer physical and mental health outcomes [1,4,5,6,7,8]. Adaptive care planning places the child/adolescent and caregiver at the center and plans for the health care encounter (i.e., hospital admission, outpatient clinic visit, surgery, procedure, or medical test) by meeting with the patient and caregiver(s) virtually in advance to plan for a hospital visit and then adapting the care required with the aim to minimize stressful experiences and provide developmental-behavioral and mental health support during the hospital visit or admission.

Adaptive care plans involve planning from home, to hospital, and back to home, while thinking of everything in between. This includes advanced planning to prepare the child/adolescent for coming to the hospital (i.e., social stories, desensitization), travel to the hospital, entry to the hospital, route to the health care encounter, the health care encounter, clustering care, when possible, and travel to home. The plans are made keeping in mind the individualized developmental, behavioral, and mental health needs of the child or adolescent using a trauma-informed approach.

Plans are made with the child/adolescent (when possible), the caregiver and the multi-disciplinary team, along with the point-of-care team and any additional care teams that may be involved or required to support or adapt their care (i.e., child life, dentistry, anesthesia, surgery). The adaptive care plan is communicated to the team caring for the child/adolescents and placed on the electronic medical record for hospital staff to use as a guide for adapting care and supporting the child/adolescents during the hospital encounter.

#### 2.4.2. Behavior Challenges

Children and adolescents with ASD/IDD may present to the hospital with behavior challenges that are impacting their physical health (i.e., self-injury, restricted eating) which require medical attention. Further, medical and health issues may be expressed through changes in behavior (i.e., constipation, pain, illness, or infection). Mental health diagnoses can also present as behavioral changes in children and adolescents with ASD/IDD. Hospital environments and medical interventions may cause significant stress leading to agitation that may be expressed through behaviors that challenge. Further, children and adolescents who have experienced medical trauma may have heightened levels of stress and anxiety in hospital settings.

A bio-psycho-social approach is used when evaluating behavioral changes for children and adolescents with ASD/IDD. A multi-disciplinary approach is vital for assessing whether a behavior change is related to an underlying health issue, a change in environment, a lack of adequate community resources and/or support for caregivers, lived experiences, life events, emotional distress and/or a psychiatric etiology [20].

All behaviors have a function, such as serving the function of communicating something, to express a want or need, to serve a sensory purpose, to seek-out social engagement, and/or to avoid an unwanted situation. Understanding the underlying reason for a behavior (i.e., to communicate pain) is important when caring for children and adolescents with ASD/IDD in a hospital setting. It can help with assessment and diagnosis and supportive behavioral strategies can be used to provide medical treatment and adapt care. A developmental-behavioral and mental health approach to providing care is important and this service provides strategies to the team for supportive behavioral care strategies that meet the individual needs of the patient (i.e., visual schedules, communication strategies, positive reinforcement).

Hospital environments can increase agitation and stress in children with ASD/IDD and can lead to agitated or aggressive behaviors that could cause harm to a patient, caregiver and/or health care staff. A key role of the developmental-behavioral and mental health service for children/adolescents with ASD/IDD is to develop developmental-behavioral and mental health plans on the electronic medical chart to help avoid behavioral agitation, de-escalation techniques and safety measures.

Some children/adolescents with ASD/IDD may require psychopharmacological intervention for behavioral or mental health presentations, including agitation, anxiety, depression and/or psychosis. Consultation surrounding psychopharmacological intervention through a multi-disciplinary approach is provided through this service.

#### 2.4.3. Developmental Regression

Children and adolescents with ASD/IDD often present to the hospital emergency department or outpatient clinic when facing a medical, neurological, mental health, behavioral, family and/or social crisis that leads to developmental regression. The cause of this regression may not be easily elucidated by caregivers or clinicians and requires a step-by-step biopsychosocial, multidisciplinary approach to assessment and treatment.

#### 2.4.4. Urgent Developmental-Behavioral-Psychiatric Care

As described above, children and adolescents with ASD/IDD may present to the emergency department at a tertiary level children’s hospital in acute or acute-on-chronic developmental-behavioral and/or mental health crisis. This multi-disciplinary team provides real-time on-call consultation to the emergency department and across the hospital and sees patients who present after hours or on weekends within 48 h to provide developmental-behavioral and/or mental health support.

### 2.5. Tertiary Level Children’s Hospital Multi-Disciplinary Care Team for Children and Adolescents with ASD/IDD

A vital aspect of this service for children and adolescents with ASD/IDD is that the team is multi-disciplinary. A unique characteristic of this initiative is that the multi-disciplinary team is comprised of specialists from a developmental center with expertise in developmental, behavioral, and mental health care with children and adolescents with ASD/IDD in partnership with health care professionals with expertise in complex medical and mental health care in a tertiary care hospital. The patient and family are considered to be vital team members and experts, at the center of the team, in a patient- and family-centered care model. An additional strength of the service is that it is integrated into existing programs and services within the hospital and works alongside point-of-care teams and other specialists to provide optimal interdisciplinary care. The multi-disciplinary team members are described below:

#### 2.5.1. Patient and Family (Family-Centered Care)

Children and youth with ASD and their families are more likely to report difficulty accessing health care services and that their care is often not family-centered [17]. In this initiative, the patient and caregiver are considered to be at the center of the multi-disciplinary care team and are vital members of a patient- and family-centered care team. Children and adolescents with ASD/IDD are included as active participants and self-advocates in their care, with a developmental approach. Caregivers are considered the expert on their child and participate in the approach to assessment, intervention, behavioral strategies, and planning. The health care team forms a collaborative partnership that places a responsive priority on the values and preferences of patients and their families. Health professionals play a key role in providing supportive counselling and education to help families make informed decisions within the team.

#### 2.5.2. Developmental Pediatrician

Developmental pediatricians have training and expertise in development, ASD/IDD, and behavioral pediatrics. On the hospital team, they provide developmental-behavioral assessment and consultation and psychopharmacological consultation. They help to understand and support behavior from a developmental, behavioral, medical, and mental health perspective.

#### 2.5.3. Child and Adolescent Psychiatrist

Child and adolescent psychiatrists have expertise in mental health assessment and diagnosis. Mental health diagnoses can often be overlooked in children and adolescents with ASD/IDD due to diagnostic overshadowing [21]. The child and adolescent psychiatrist requires specialized training and interest in mental health of children and adolescents with ASD/IDD. They help to diagnose and treat mental health symptoms in children with ASD/IDD. Further, they provide psychopharmacological consultation. Finally, they provide family centered treatment and therapy, which is often a vital aspect of a child or adolescent’s care plan.

#### 2.5.4. General Pediatrician

General pediatricians with expertise in development and mental health are part of the team. Many children at a tertiary level children’s hospital have complex medical, developmental, and mental health needs and general pediatricians provide expert medical care. Their role is to provide in-depth medical assessment and management in collaboration with the developmental pediatrician and psychiatrist.

#### 2.5.5. Behavior Analyst

While few hospitals have Board Certified Behavioral Analysts (BCBA), a behavioral expert is considered vital to providing optimal developmental-behavioral, mental health and medical care to children with ASD/IDD in a tertiary children’s hospital. There is emerging evidence showing the need for BCBAs or similar professionals with behavioral expertise for supporting behavior during hospitalizations for children and adolescents with ASD/IDD [22]. The BCBA assesses behavior, analyzes the function of the behavior(s), and provides behavioral assessment information that can help to delineate medical, behavioral and/or mental health presentations. Further, they develop behavioral plans, safety plans, and provide consultation on behavior strategies to support children and adolescents while in hospital or undergoing medical procedures or treatments. If a child is started on a psychopharmacological intervention, behavior analysts can collect and analyze data to help assess if a medication is effective.

Behavior strategies and plans, such as those used in Positive Behavior Support [23], are integrated within the inter-disciplinary model and these strategies are encouraged to be used by all members of the health care team caring for the patient throughout the hospital setting.

#### 2.5.6. Developmental Service Worker

Developmental service workers have experience in working one-on-one with children and adolescents with ASD/IDD. They support the BCBA in data collection. They help to carry out and implement the multi-disciplinary developmental-behavioral and mental health care plans at the bedside. Along with the BCBA, physicians and the multi-disciplinary team, they also involve the family in the intervention goals and help caregivers learn the strategies and apply them within the hospital setting, as able, in order to help with generalization of these behavior and developmental support strategies in the home setting and within the family context.

#### 2.5.7. Developmental Service Coordinator

A vital part of developmental-behavioral and mental health care is accessing community resources, funding and supports. Many children, adolescents and caregivers who present to the hospital are lacking adequate resources and supports. Often this is contributing to their developmental, behavioral, medical, or mental health challenges. The developmental service coordinator is vital in supporting families to ensure they are accessing resources, funding, school supports, and supports in the community from social service agencies, in addition to health agencies. They also help develop discharge intervention plans and ensure supports are in place when patients leave the hospital. Getting adequate medical, developmental, behavioral, and mental health supports and resources in place helps to avoid re-presentation to the emergency department and hospital.

#### 2.5.8. Nursing

Nurses with expertise in working with children and adolescents with ASD/IDD provide nursing support to the team. They have expertise in adapting medical care for patients with ASD/IDD (i.e., taking vitals, giving medications) and prevention and de-escalation strategies for patient distress or agitation. They play a key role in adaptive care planning and coordination of adaptive care plans. They also help to build capacity among other nurses and health care professionals to support children and adolescents with ASD/IDD across the hospital. Between appointments, they are the main point of contact for families to reach out to for help.

#### 2.5.9. Operations Manager

The operational planning and administration for a busy multi-disciplinary service requires an operational manager to support the operations, program development, human resources and vision for innovation and growth of the team.

#### 2.5.10. Program Coordinator

The program coordinator coordinates the clinics, clinician’s schedules, patient scheduling and learner’s schedules as well as supports the daily operations of the multi-disciplinary team. Further, the program coordinator collects program data and statistics for reporting and continuous quality improvement and program development.

#### 2.5.11. Integration with the Point-of-Care Team and Existing Hospital Services

Along with a close collaboration with the point-of-care team, this service works as a multidisciplinary team with many existing hospital services. Among these are the point-of-care team (i.e., the most responsible physician’s team, such as general pediatrics, oncology, dentistry, anesthesia, and surgical subspecialties) along with other services such as social work, child life, occupational therapy, speech therapy, dieticians, phlebotomy, and the Code White team). The integration of this team within the hospital, along with close interprofessional collaboration with existing hospital services and specialists, strengthens the supports and adaptive care that can be provided as a team to the patient, and family. Further, the integration and interprofessional team interactions can help to build capacity among health care staff within the hospital.

## 3. Discussion

Children and adolescents with a diagnosis of ASD/IDD experience greater barriers in accessing health care and are at risk of experiencing poorer physical and mental health outcomes than their peers without ASD/IDD. Hospital environments and health care encounters can be stressful and overwhelming for children and adolescents with ASD/IDD, especially when individualized supports are not in place. In addition, hospital medical staff, who are largely trained and experienced in assessment and management of acute, complex, and chronic medical needs of patients may feel unequipped for the behavioral and mental health needs of children and adolescents with ASD/IDD. This paper identifies the gaps in care for children and adolescents with ASD/IDD in a tertiary level children’s hospital and highlights the need for a hospital-based specialized multi-disciplinary developmental-behavioral and mental health care team. It provides a general framework for developing a model of care to support patients with ASD/IDD along with clinical care pathways for supporting the developmental-behavioral and mental health of children and adolescents with ASD/IDD in a hospital setting. It also describes the multidisciplinary team members essential to supporting children and adolescents in a tertiary care children’s hospital and the importance of integrating with current services and teams.

A strength of this model is that it is a collaborative initiative between a center with developmental-behavioral and mental health expertise and is integrated within the existing services in a tertiary children’s hospital, with each center bringing unique skills to support children and adolescents with ASD/IDD who require tertiary level medical care. Further, through integrated, interprofessional care, this model may help to build capacity among hospital staff and learners.

While many school boards have specialized multi-disciplinary teams to support students with ASD/IDD and provide consultation to the teachers and staff to help support learning and behavior, most hospitals do not have similar specialized teams dedicated to providing developmental-behavioral and mental health support for patients with ASD/IDD and consultation to hospital staff. The literature suggests that there is a need for hospital-based developmental-behavioral expertise and training to support hospitalized children and adolescents with ASD/IDD [8]. This model of care is an innovative approach that aims to fill-in those gaps through a multi-disciplinary approach to care that is adapted to meet each patient’s individual needs. Adapting care and providing behavioral support can help children and adolescents with ASD/IDD access medical, developmental, and mental health care in a timely manner and create a safe and supportive hospital environment.

Understanding behavior is important in the health care setting. For some children and adolescents with ASD/IDD, behavior may represent the communication of pain or an underlying medical or mental health condition. In this integrated multi-disciplinary service model, the team can assess the medical, social/environmental, mental health and behavioral needs of a patient through a comprehensive bio-psycho-social approach for more timely and accurate diagnoses and treatment plans.

Behavior and mental health challenges may also create a barrier to accessing medically necessary care. Through a specialized developmental-behavioral and mental health care team approach, medical care can be adapted and behavioral strategies and/or psychopharmacological interventions, when needed, can be set in place to help patients access the tertiary level medical care they need.

This initiative aims to improve care, decrease barriers and health care inequities, and ultimately improve long-term physical and mental health outcomes in individuals with ASD/IDD through a Plan-Do-Study-Act (PDSA) model of health care quality improvement [24]. This paper describes the P-planning and D-doing of the PDSA cycle for improving quality of care. Future directions will be to study the outcomes of the impact of this initiative. Specifically, it will be important to S-study physical and mental health outcomes, including number or number of visits to the emergency department, hospitalizations, length of stay, level of distress with health care encounters, patient, caregiver and hospital staff satisfaction with the service, safety outcomes, number of code whites, and assessment of whether this integrated model helps to build capacity for caring for children with ASD/IDD among hospital staff and learners. Further, measures of quality of life to assess wellbeing in children and adolescents with ASD/IDD cared for in this program, with a rights-based approach in mind [25], will also be important to study. Based on these analyses, this model will be improved through A-acting on what is learned and continuing to improve the supports for children and adolescents with ASD/IDD within the tertiary hospital setting.

## Figures and Tables

**Table 1:** Stakeholder engagement discussion questions.

Questions
What do you feel are the greatest gaps in care/unmet needs for children with ASD/IDD at the hospital?
Which patients with ASD/IDD at the hospital require a team with specialized expertise the most (what do you feel should be the priorities)? How can we best collaborate with you and other relevant services in the hospital? Are there areas in which we could integrate within current services to strengthen care delivery? What ideas do you have on how we could measure success? What would be the most relevant outcomes in your opinion?

**Table 2:** Stakeholder perspectives on tertiary level hospital care needs for children and adolescents with ASD/IDD.

Themes
There is a significant need for developmental/behavioral pediatric expertise, system-wide, at a tertiary level children’s hospital. A tertiary level children’s hospital would greatly benefit from more specialized, comprehensive, cohesive and interprofessional developmental-behavioral pediatric care. There is a need for coordinated interprofessional care for children and adolescents with ASD/IDD at a tertiary level children’s hospital. More support is needed for children and adolescents presenting to a tertiary level children’s hospital emergency department in acute or acute-on-chronic crisis due to escalated behavior challenges and/or extensive support needs. There is a need for a program with a system-wide approach to supporting children and adolescents with ASD/IDD to decrease presentations/repeated presentations to the emergency department and hospital admissions. More behavioral support is needed for inpatients with ASD/IDD, along with support for their caregivers and consultation to hospital staff. Some of the patients with ASD/IDD currently seen by other specialties/services in a tertiary level children’s hospital may be better served collaboratively along with a specialized developmental pediatric, psychiatry and behavior support team.
There is a need to build capacity among health care professionals and learners to develop behavioral-medicine expertise and skills for caring for children and adolescents with ASD/IDD in a tertiary level children’s hospital setting. Specialized developmental-behavioral care aligns with the current tertiary level hospital vision for building hospital-wide mental health supports. A tertiary level specialized developmental-behavioral hospital care service should be multi-disciplinary and interprofessional. ○ It should be integrated within current services to fill-in the gaps that exist for developmental-behavioral supports for children and adolescents with ASD/IDD. ○ It should work collaboratively as an interprofessional team with Child and Adolescent Psychiatry. Adaptive care planning is recommended to help children and adolescents with ASD/IDD access medically necessary care in a timely and safe manner and to minimize stressors associated with hospital encounters. ○ Care should be individualized and adapted to meet the specific needs of the child/adolescent and family. A standardized, interprofessional, bio-psycho-social approach is recommended for diagnosis and management of developmental/behavioral regression in children and adolescents with ASD/IDD.
